# Comparison of the Influence of 45S5 and Cu-Containing 45S5 Bioactive Glass (BG) on the Biological Properties of Novel Polyhydroxyalkanoate (PHA)/BG Composites

**DOI:** 10.3390/ma13112607

**Published:** 2020-06-08

**Authors:** Katharina Schuhladen, Barbara Lukasiewicz, Pooja Basnett, Ipsita Roy, Aldo R. Boccaccini

**Affiliations:** 1 Department of Materials Science and Engineering, Institute of Biomaterials, University of Erlangen-Nuremberg, 91058 Erlangen, Germany; katharina.ks.schuhladen@fau.de; 2 Applied Biotechnology Research Group, School of Life Sciences, College of Liberal Arts and Sciences, University of Westminster, London W1W 6UW, UK; barbara.lukasiewicz@gmail.com (B.L.); P.Basnett@westminster.ac.uk (P.B.); 3 Department of Material Science and Engineering, Faculty of Engineering, University of Sheffield, Mappin Street, Sheffield S1 3JD, UK

**Keywords:** bioactive glass, copper, polyhydroxyalkanoates, tissue engineering, scaffolds

## Abstract

Polyhydroxyalkanoates (PHAs), due to their biodegradable and biocompatible nature and their ability to be formed in complex structures, are excellent candidates for fabricating scaffolds used in tissue engineering. By introducing inorganic compounds, such as bioactive glasses (BGs), the bioactive properties of PHAs can be further improved. In addition to their outstanding bioactivity, BGs can be additionally doped with biological ions, which in turn extend the functionality of the BG-PHA composite. Here, different PHAs were combined with 45S5 BG, which was additionally doped with copper in order to introduce antibacterial and angiogenic properties. The resulting composite was used to produce scaffolds by the salt leaching technique. By performing indirect cell biology tests using stromal cells, a dose-depending effect of the dissolution products released from the BG-PHA scaffolds could be found. In low concentrations, no toxic effect was found. Moreover, in higher concentrations, a minor reduction of cell viability combined with a major increase in VEGF release was measured. This result indicates that the fabricated composite scaffolds are suitable candidates for applications in soft and hard tissue engineering. However, more in-depth studies are necessary to fully understand the release kinetics and the resulting long-term effects of the BG-PHA composites.

## 1. Introduction

Due to disease, injury and trauma, treatments to promote the repair, replacement or regeneration of damaged and degenerated tissues in the human body are necessary. These treatments typically involve living tissue and organs for transplantation and have been lifesaving [1,2,3,4]. However, due to donor limitations and organ rejection, tissue engineering (TE) as a suitable alternative is being increasingly investigated [5]. In the most common TE approach, cells grow on a scaffold made using suitable methods to provide a temporary support and a well-defined pore structure [6]. Furthermore, growth factors and other biomolecules can be incorporated within the scaffold in order to guide the regulation of cellular functions during tissue regeneration [7]. The first important step in the designing of TE scaffolds is to find suitable biomaterials for building a 3D structure, which would degrade appropriately, in a rate similar to the new tissue growth rate. Natural and synthetic polymers are widely used as biomaterials for TE scaffolds [8]. Natural polymers are extracted from animals or plants (e.g., collagen, chitosan), whereas synthetic polymers are synthesized chemically (e.g., poly-L-lactic acid, poly-ε-caprolactone) [9].

Polyhydroxyalkanoates (PHAs) are polyesters of 3-, 4-, 5- and 6-hydroxyalkanoic acid which are synthesized using microbial biotechnology approaches involving bacterial fermentation. Some bacteria store PHAs as an intracellular storage compounds for energy and carbon, normally under conditions of nutrient limitation (e.g., nitrogen, sulphur, oxygen, magnesium or phosphorus), with the excess of carbon (e.g., carbohydrates, lipids, fatty acids) [10,11,12,13]. The number of carbon atoms in a monomer unit is crucial to the properties of PHAs. Depending on this number, PHAs can be classified as short-chain length PHAs (scl-PHAs) and medium-chain length PHAs (mcl-PHAs). Scl-PHAs contain 3–5 carbon atoms, whereas mcl-PHAs contain 6–14 carbon atoms. Scl-PHAs are generally brittle and stiff, with a high melting point and crystallinity, except for poly(4-hydroxybutyrate), P(4HB). Typical examples for scl-PHAs include poly(3-hydroxybutyrate-co-3-hydroxyvalerate), P(3HB-*co*-3HV), and the most well studied one, poly(3-hydroxybutyrate), P(3HB) [14]. Mcl-PHAs, on the other hand, are semi-crystalline polymers, have a low melting point, and are extremely elastomeric. Poly(3-hydroxyhexanoate) and poly(3-hydroxyoctanoate) are typical examples for mcl-PHAs [10,15,16]. In general, PHAs are biodegradable, insoluble in water, nontoxic, biocompatible, piezo-electric (which stimulates bone growth and promotes wound healing), thermoplastic and/or elastomeric [15,16,17].

Polymers, such as PHAs, are highly biocompatible and can be easily formed in complex shapes and structures. However, in order to further enhance their biological properties and to tailor the properties of PHAs for different applications, these biomaterials can be mixed with inorganic components (e.g., hydroxyapatite, bioactive glasses) forming composites [9,18,19]. For example, such composites have been shown to have enhanced capability to form an apatite layer on the implant surface, which for instance is important in the regeneration of bone [20,21,22].

In 1969, Hench and co-workers invented the first bioactive glass (BG), showing a strong bone bonding ability of 45S5 BG [23]. This glass composition (45% SiO_2_-24.5% Na_2_O-24.5 CaO-6% P_2_O_5_ in wt.%) is highly reactive when in contact with an aqueous environment [23,24]. It has been reported that 45S5 BG has been used in more than a million patients to repair bone defects in the jaw and in orthopaedics [24]. The 45S5 BG is in general bioactive, biocompatible, biodegradable, osteoconductive, osteoinductive, angiogenic, nontoxic and noninflammatory because of its ability to form a hydroxycarbonate apatite surface layer in a biological medium and exploiting the release of ionic dissolution products that stimulate specific cellular pathways [24,25,26,27,28,29]. Since the release of ionic dissolution products, after exposure to a physiological environment, is believed to improve the bioactivity of materials, new approaches for enhancing BG bioactivity are being investigated by introducing therapeutic ions in BG compositions [30,31,32]. In this work, Cu-doped BG was used as an example of the use of inorganic ions to further enhance the bioactivity of PHAs. Copper has been shown to promote synergistically stimulating effects on angiogenesis by stabilizing the expression of hypoxia-inducible factors and promote the proliferation of human endothelial cells [33,34,35,36,37,38]. Moreover, Cu ions were shown to promote wound healing in rats, which has been linked to the up regulation of vascular endothelial growth factor (VEGF) by stimulated cells [6] and to the antimicrobial behavior of Cu. Indeed, Cu is able to artificially mimic hypoxia, which plays an important role in blood vessel formation as well as in the differentiation and recruitment of endothelial cells [37,39]. The angiogenic effect of Cu-doped 45S5 BG has been investigated and proven *in vitro* and *in vivo* using 3D BG scaffolds [40].

In the present work, 3D mcl/scl-PHA scaffolds containing 45S5 and Cu-doped 45S5 BGs were prepared using solvent casting/salt leaching technique in order to create an advanced PHAs based composite with Cu-ion delivery capability, and with tailored properties for use in different types of hard and soft tissue engineering. For the first time, the interaction of stromal (ST2) cells with the dissolution products of the neat and composite PHA scaffolds was studied by evaluating the cell viability, cell morphology and the release of VEGF from the cells cultivated in the presence of dissolution products from the scaffolds. The newly designed composites are considered to be suitable for soft and hard tissue engineering due to the enhanced angiogenic effect potentially due to the release of therapeutic ions, especially copper, from the BG particles embedded in the PHA matrix of the scaffold [10,16,23,24].

## 2. Materials and Methods 

### 2.1. Materials

All the chemicals used for PHA production were purchased from VWR (Lutterworth, UK) or from Sigma-Aldrich (Dorset, UK). The two different BGs, the 45S5 composition (in wt.%: 45.0 SiO_2_, 24.5 Na_2_O, 24.5 CaO, 6 P_2_O_5_) and a Cu-doped 45S5 composition (in wt.%: 45.0 SiO_2_, 24.5 Na_2_O, 22.0 CaO, 6 P_2_O_5_, 2.5 CuO) used in this study were produced by melt-quenching, presented elsewhere [40]. Briefly, the BGs were produced by mixing SiO_2_, Na_2_CO_3_, CaCO_3_, Ca_3_(PO_4_)_2_ and CuCO_3_·Cu(OH)_2_ (all analytical grade) and melting the raw materials in a platinum crucible at 1450 °C for 45 min. The produced BGs were milled using a jaw crusher and a planetary mill (both Retsch, Germany) to obtain a particle size of d_50_ = 5 µm.

### 2.2. Scl-PHA Production

A P(3HB) polymer was synthesized by using Gram-positive bacterium *Bacillus subtilis* OK2. The production with some modifications was carried out as described elsewhere [11], by using a modified seed culture medium and a modified Kannan and Rehacek medium. Briefly, a single colony of this strain was grown at 30 °C and 200 rpm for 16 h in a seed culture medium (containing (g/L): meat peptone, 4.3; casein peptone, 4.3; sodium chloride, 6.4). Afterwards, the inoculum was used to inoculate a fermenter containing the modified Kannan and Rehacek medium (containing (g/L): glucose, 35; yeast extract, 2.5; ammonium sulphate, 5.0, potassium chloride, 3.0). The culture was grown for 48 h with constant stirring (200 rpm) and an air flow (1 L of air/min/1 L of media) at 30 °C. 

### 2.3. Mcl-PHA Production

P(3HO), P(3HO-*co*-3HD) and P(3HO-*co*-3HD-*co*-3HDD) polymers were produced using Gram-negative bacterium *Pseudomonas mendocina* CH50, obtained from the National Collection of the Industrial and Marine Bacteria (NCIMB 10541) and different carbon sources (Table 1) based on optimized protocols [41,42,43]. A single colony of this strain was grown under the same conditions and in the same seed culture medium as described for P(3HB). The remaining production steps for mcl-PHAs were described in Rai et al. [43]. Briefly, to inoculate the second stage, a modified mineral salt medium (containing (g/L): ammonium sulphate, 0.45; potassium phosphate monobasic, 2.38; Di-sodium hydrogen phosphate anhydrous, 3.42; magnesium sulphate heptahydrate, 0.8; trace element solution, 1 mL/L; suitable carbon sources, (Table 1)) and to inoculate the production stage, a second modified mineral salt medium (containing (g/L): ammonium sulphate, 0.5; potassium phosphate monobasic, 2.65; Di-sodium hydrogen phosphate anhydrous, 3.8; magnesium sulphate heptahydrate, 0.8; trace element solution, 1 mL/L; related carbon sources, (Table 1)) were used. In order to simplify the labelling of the different tested PHAs, the short versions of the mcl-PHAs were used according to Table 1.

### 2.4. PHA Extraction

For the extraction of PHAs, the cells were harvested by centrifuging the cultures at 4600 rpm, then homogenized and lyophilized. In order to extract the polymer, Soxhlet extraction was used firstly with methanol and secondly with chloroform. The extracted solution was concentrated by evaporation, followed by the precipitation of the polymer, using chilled methanol with continuous stirring [43].

### 2.5. PHA and BG-PHA Scaffold Preparation

The PHA and BG-PHA scaffolds were prepared by using the salt-leaching technique. For composite scaffolds, 1 wt.% (in case of scl-PHAs) or 2 wt.% (in case of mcl-PHAs) of 45S5 BG/45S5-2.5Cu powder was added to the dissolved PHAs (to obtain a final BG content of 20 wt.%) in chloroform (5 wt.% of scl-PHAs/10 wt.% of mcl-PHAs) and dispersed by sonication. Sodium chloride (sieved, diameter, 355 µm, Sigma Aldrich, Dorset, UK) was then added in the 10:1 ratio to the PHAs, as a porogen. The solution was cast layer by layer in a mould (3.5 cm × 1.2 cm). After drying, the scaffolds were immersed in distilled water for 24 h in order to dissolve the sodium chloride, and thus to form pores [44].

### 2.6. Scanning Electron Microscopy (SEM)

Characterization of the scaffolds was carried out using a scanning electron microscope (LEO 435 VP; LEO Electron Microscopy Ltd., Cambridge, UK, and Ultra Plus; Zeiss, Jena, Germany). The samples were placed on the 8 mm diameter aluminum holder and images were then recorded at different magnifications. The microscope was operated at 1 kV and a working distance of 2 mm. The pore size range was calculated based on at least 5 different SEM images per sample (not shown here) using ImageJ (NIH, Bethesda, MD, USA) [45].

### 2.7. Cell Culture

Stromal ST2 cells (Deutsche Sammlung von Mikroorganismen and Zellkulturen GmbH, Braunschweig, Germany) derived from the mouse bone marrow of BC8 mice were used to study the biocompatibility of the produced scaffolds. ST2 cells were chosen due to their potential to differentiate in osteoblasts, adipocytes and hematopoietic cells [46]. The cells were grown, harvested and counted as described by Balasubramanian et al. [47]. For the biocompatibility test, 100,000 ST2 cells per mL of cell culture medium (CCM, RPMI 1640 medium (Gibco, Schwerte, Germany) containing 10 vol.% fetal bovine serum (Sigma-Aldrich, Darmstadt, Germany) and 1 vol.% penicillin/streptomycin (Gibco, Germany)) were transferred per well in a 48-well plate (VWR, Darmstadt, Germany) and incubated for 24 h. Scaffolds were cut in 0.1 ± 0.01 g cubes and disinfected by using UV for a period of 30 min on each side. The scaffolds were immersed in 1 mL of CCM and incubated for 24 h under the same conditions as the cells. After 24 h, 1 mL CCM containing ionic dissolution products (IDPs) of 0.1 ± 0.01 g of scaffolds was removed and named 10%-CCM in accordance to a previous publication [45] (although the 0.1 g of scaffold did not completely dissolve). By further diluting this 10%-CCM, dilutions were produced and named as 1%-, 0.1%- and 0.01%-CCM in order to simplify the labelling. After removing the CCM from the cells incubated for 24 h, the now attached ST2 cells were incubated with the different dilutions of the CCM containing IDPs for 48 h under the same conditions as described above. Cells grown in CCM containing no IDPs were taken as a reference. Every sample type was investigated as replicates of three. After 48 h, the CCM was collected in Eppendorf tubes for VEGF release measurement studies. The cell viability using a WST-8 assay (Sigma Aldrich) and the release of VEGF was tested as described by Balasubramanian et al. [47]. Briefly, the VEGF release was measured by using a RayBio mouse VEGF enzyme-linked immunosorbent assay (ELISA) kit. The cell viability, as well as the release of VEGF, was determined by following the manufacturer’s protocol and then spectrometrically analyzed using a microplate reader (PHOmo, Anthos Mikrosysteme, Krefeld, Germany) at 450 nm. Further, hematoxylin and eosin (H&E) staining was used to investigate the morphology. Prior to staining, cells were washed with PBS and fixed using Fluoro-fix. After another washing step with distilled water, the cells were stained using Hematoxylin for 10 min. Subsequently afterwards, Hematoxylin was removed by first washing with tap water, followed by “Scott’s tap water” and distilled water for 1–5 min. Then, cells stained with an Eosin solution (0.1% Eosin in 90% ethanol and 5% acetic acid) for 5 min and finally washed with 100% ethanol. Stained cells were observed using a light microscope (Primo Vert, Zeiss, Oberkochen, Germany).

## 3. Results

**Figure 1:**
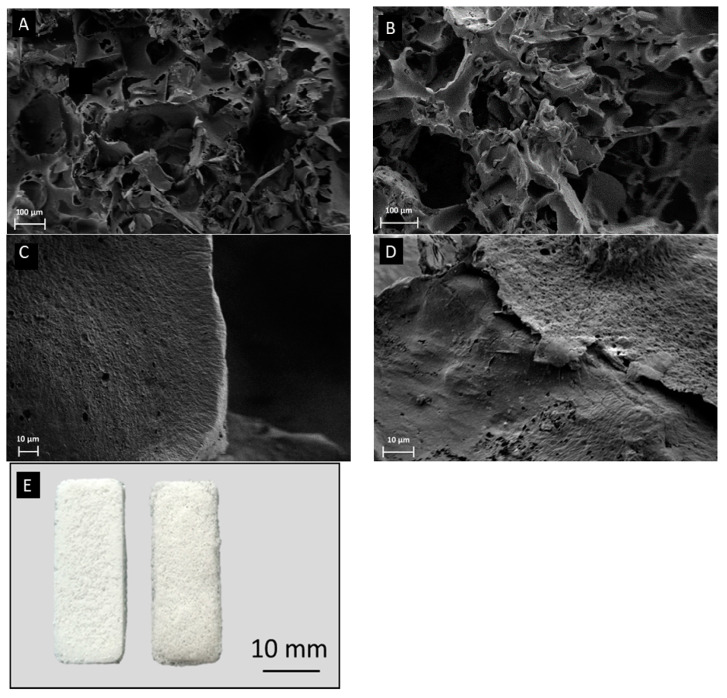
SEM images of neat P(3HB) scaffold (**A**), neat P(3HO) scaffolds (**B**), P(3HB)/Cu-doped bioactive glass (BG) composite (**C**), and P(3HO)/Cu-doped BG composite (**D**). (**E**) Digital camera images of P(3HB)/BG (left) and P(3HO)/BG (right) scaffolds.

In Figure 1, SEM images of neat and composite P(3HB) and P(3HO) scaffolds can be seen. The pores were of irregular shape and of varied sizes (10–250 µm) proven by the SEM images in Figure 1A,B. Moreover, good pore interconnectivity was indicated by the SEM images, which needs to be proven in future work (e.g., by µCT). The fabrication technique used to prepare these samples leads to results similar to the ones achieved for other polymers using this method [48]. No obvious difference could be observed between the neat scl-PHAs and mcl-PHAs scaffolds. Furthermore, it was confirmed that the microstructures of the composite foams were similar to that of the neat ones. In Figure 1, SEM images of neat P(3HB) (C) and a composite P(3HB) (D) scaffold at high magnification are shown as examples. No BG particles could be found on the surface of the composite P(3HB) scaffolds, and the same was observed for all composite mcl-PHAs scaffolds. This observation confirmed that the BG particles were fully embedded in the polymer matrix. Figure 1E shows an exemplary digital image of a P(3HB)/BG and a P(3HO)/BG scaffold, indicating that the scaffolds maintained the shape of the used molds.

**Figure 2:**
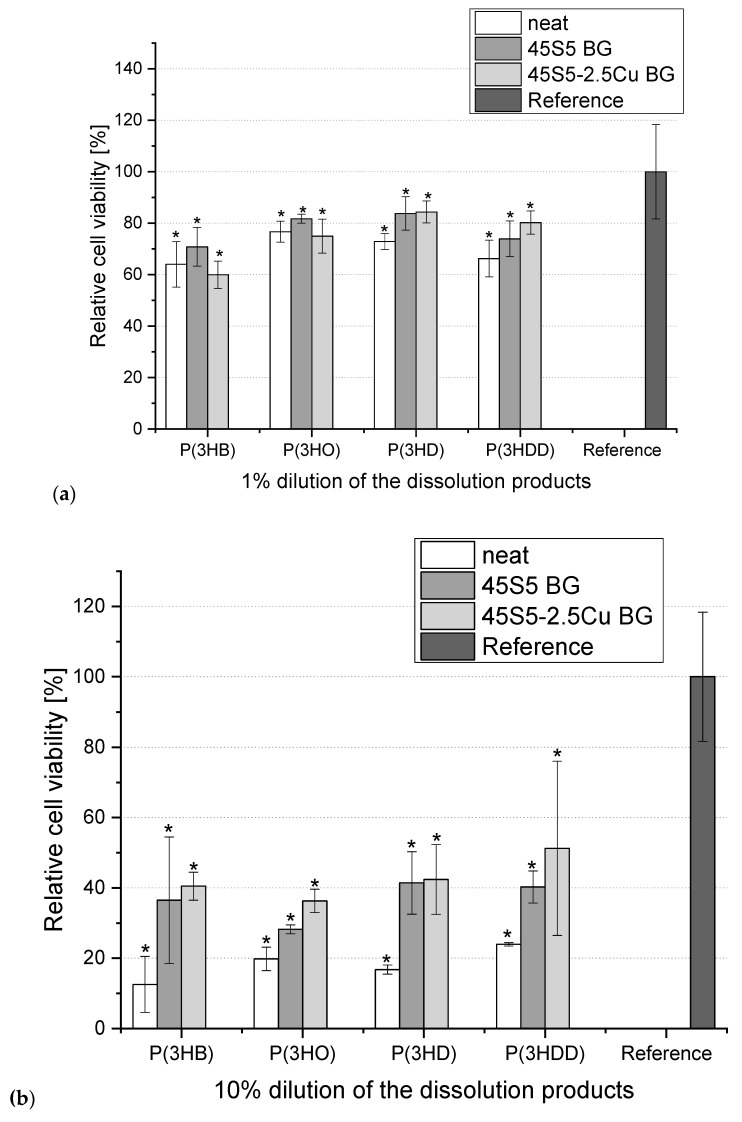
Relative viability of ST2 cells cultured in media containing 1% (**a**) and 10% (**b**) dilution of ionic dissolution products (IDPs) from different BG-PHA and neat PHA scaffolds. One-way ANOVA statistical analysis denotes significant differences compared to the reference (* *p* < 0.05). As reference pure CCM was used (Bonferroni’s post hoc test was used).

There were no differences between the viability of cells grown in 0.01%-CCM and 0.1%-CCM of all the different scaffolds compared to the cells grown in CCM without any IDPs, and Figure 2 shows the results for the cells grown in 1%- and 10%-CCM. As seen in Figure 2a, a 60–80% decrease in cell viability was observed when 1%-CCM was added as compared to the reference for both the neat and composite PHA samples. No significant difference was found between the different PHAs. Furthermore, the addition of BG did not seem to have any influence. Figure 2b shows that cells grown in 10%-CCM exhibited remarkably less cell viability. Here too, no significant influence of adding BG into the polymer scaffold was detected.

**Figure 3:**
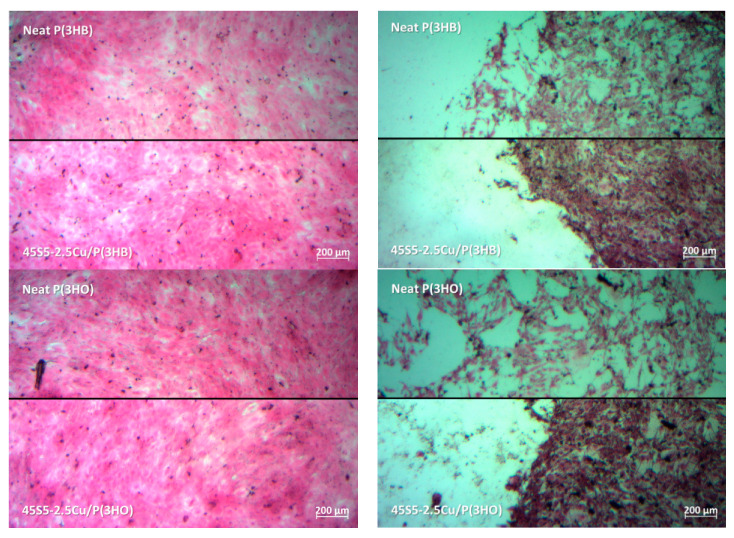

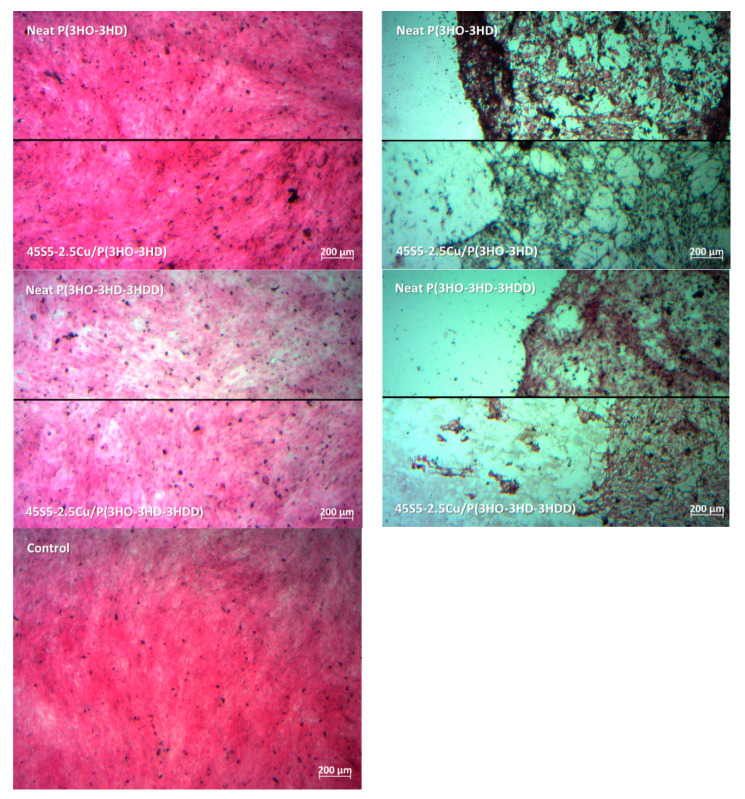
Images of hematoxylin and eosin (H&E)-stained cells cultured in 1% (left) and in 10% (right) dilution of the dissolution products of the different neat and composite PHA scaffolds. Cells cultured in CCM containing no dissolution products were used as control.

Light microscopy images of H&E-stained ST2 cells cultured with 1%-CCM and 10%-CCM of neat PHA scaffolds and PHA scaffolds containing 45S5 BG are shown in Figure 3. Cells cultured in 1%-CCM of BG-PHA and neat PHA scaffolds exhibited their typical phenotypic cell morphology, and showed adhesion to the well plate. Moreover, the density of cells grown on 1%-CCM was seen to be almost the same as the density observed in the reference sample (control). This indicates that ST2 cells grown in contact with the dissolution products of the PHA scaffolds containing additional BG in concentrations lower than 10% do not have any adverse effect on the cell morphology. However, cells cultured in 10%-CCM of all scaffolds showed relatively poor cell proliferation and adhesion. These results confirm the data from the cell viability test. According to Figure 3, in all CCM containing the same concentration of IDPs, only minor differences were observed between the different PHAs as well as between the neat and BG-containing scaffolds. 

**Figure 4:**
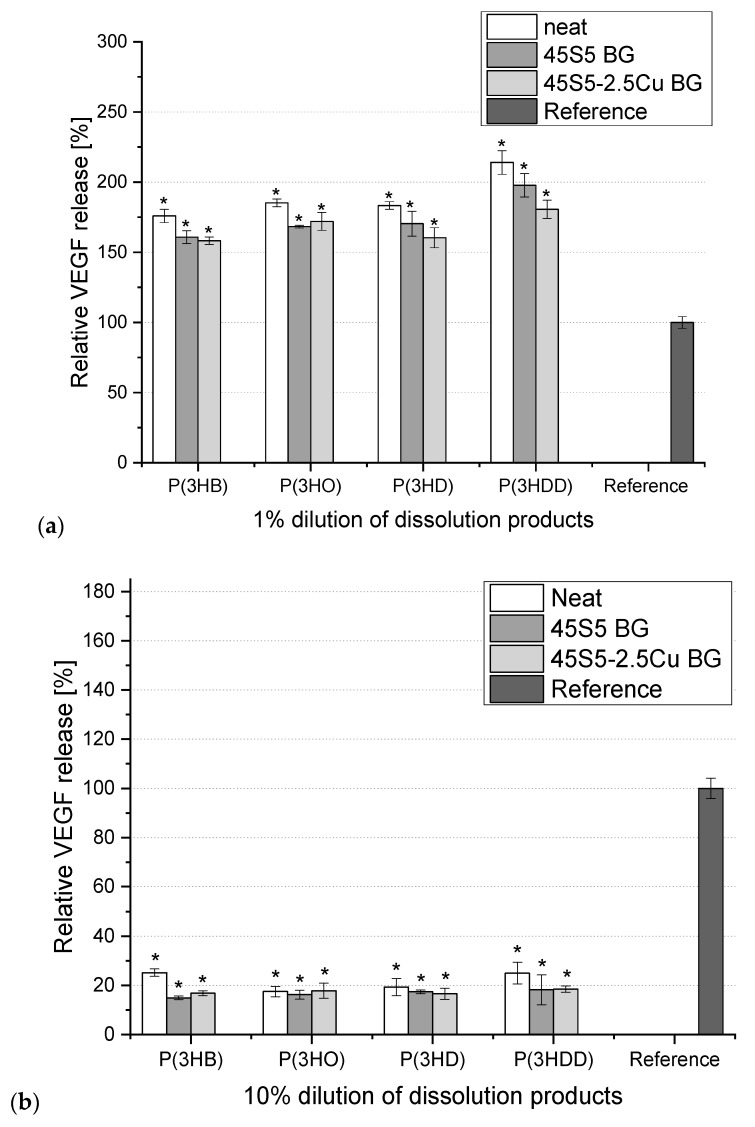
Relative vascular endothelial growth factor (VEGF) release from ST2 cells cultured in media containing 1% (**a**) and 10% (**b**) dilution of dissolution products of BG-PHA and neat PHA scaffolds. One-way ANOVA statistical analysis denotes significant difference (* *p* < 0.05) compared with the reference. As reference pure CCM was used (Bonferroni’s post hoc test was used).

The VEGF release from the ST2 cells cultured in media with different dilutions (10, 1, 0.1 and 0.01%) of dissolution products of different neat and composite PHA scaffolds was measured. Since no difference between the VEGF release from cells cultured in media containing 0.1%-CCM and 0.01%-CCM of all BG-PHA and neat PHA scaffolds compared to the reference could be observed, these results are not shown here. According to Figure 4a, it was observed that the VEGF release increased, up to 150–220%, for all the investigated scaffolds. In contrast to cells grown in 1%-CCM, VEGF released by cells cultured in the 10%-CCM of all other neat and composite scaffolds exhibited a major reduction. In summary, the cells cultured in 1%-CCM showed a minor reduction of cell viability, but the remaining cells released higher concentrations of VEGF, whereas cells grown in 10%-CCM were relatively less viable and able to release less VEGF as compared to the cells cultured in 1%-CCM. 

Overall, the promotion of the bioactivity by adding bioactive glass in the case of bioactive glass/PHA scaffolds could only be found in the case of 1%-CCM. Here, the addition of bioactive glass led to a major increase in VEGF release and to a minor increase in cell viability. Several studies in the past have already proven the biocompatibility of different PHAs [16]. Additionally this study indicates that the polymer can promote VEGF secretion. Angiogenesis plays a crucial role in tissue regeneration and therefore is essential for the success of scaffolds in tissue engineering [28]. VEGF is known to be involved in the formation of blood vessels, and therefore the ability of PHAs to increase VEGF secretion makes them interesting for applications in tissue engineering. However, in the case of 10% dilution, the dissolution products of all scaffolds showed a major reduction of cell viability and VEGF release, proven by the cell morphology. A possible reason could be that the used salt to produce the scaffolds was not completely leached out and could therefore have a negative impact on cell viability and VEGF release. Additionally, it is also possible that the lower concentration of the 1% dilution leads to the measured increase in VEGF release as observed in a previous study [49]. Moreover, the 0.01% and 0.1% dilutions showed no influence on cell viability and VEGF release compared to the reference. Therefore, the concentration of the CCM seems to play a crucial role and further studies, especially under dynamic conditions, are necessary to understand the dose-dependent effect on VEGF release. A significant effect of the addition of BGs in the polymer matrix could not be measured. It is well known that the degradation and therefore the dissolution of ions start from the surface of bioactive glass particles [6], hence it is important that bioactive glass particles are exposed to the fluid environment at various stages during the *in vitro* degradation of the composite. Here, most of the BG was not present on the surfaces of the scaffolds, which was proven by SEM. Instead, BG particles were embedded in the polymer matrix and therefore prevented from direct contact with the CCM. Therefore, it is suggested that an immersion time of 24 h is not enough to measure any significant release of BG dissolution products and their influence on cell viability and VEGF secretion. Moreover, future experiments should include direct cell biology studies in order to test the impact that the stiffness and the topography of the BG-PHA scaffolds have oncell viability and VEGF secretion.

## 4. Conclusions

In this study, composite BG-PHA scaffolds were successfully produced using the salt leaching technique. To evaluate the biocompatibility and the effect of the addition of BG, cell studies on the fabricated scaffolds were performed. The results indicated good biocompatibility and thus the present composites are interesting for applications in tissue engineering. However, long-term cell studies need to be carried out to better examine the effect of dissolution products of BGs, which should be followed by *in vivo* studies.

## Figures and Tables

**Table 1:** Produced mcl-PHAs with the related carbon sources.

Mcl-PHA	Abbreviations	Carbon Source	Concentration
P(3HO)	P(3HO)	Sodium octanoate	3.36 g/L
P(3HO-3HD)	P(3HD)	Glucose	20 g/L
P(3HO-3HD-3HDD)	P(3HDD)	Coconut oil	20 g/L
